# Neuropsychiatric symptoms in siblings of children with Tourette syndrome in the EMTICS study

**DOI:** 10.1002/jcv2.12277

**Published:** 2024-09-20

**Authors:** Olga Sidiropoulou, Jennifer Glaus, Julie Hagstrøm, Setareh Ranjbar, Renata Rizzo, EMTICS collaborative group, Pieter J. Hoekstra, Andrea Dietrich, Kerstin J. Plessen

**Affiliations:** ^1^ Division of Child and Adolescent Psychiatry Department of Psychiatry Lausanne University Hospital and University of Lausanne Lausanne Switzerland; ^2^ Child and Adolescent Mental Health Center Copenhagen University Hospital – Mental Health Services CPH Copenhagen Denmark; ^3^ Center for Research in Psychiatric Epidemiology and Psychopathology Department of Psychiatry Lausanne University Hospital and University of Lausanne Lausanne Switzerland; ^4^ Department of Clinical and Experimental Medicine Child and Adolescent Neurology and Psychiatry Catania University Catania Italy; ^5^ Department of Child and Adolescent Psychiatry University Medical Center Groningen University of Groningen Groningen The Netherlands; ^6^ Accare Child Study Center Groningen The Netherlands; ^7^ Faculty of Biology and Medicine University of Lausanne Lausanne Switzerland

**Keywords:** ADHD, autism spectrum disorder, ODD, siblings, Tourette syndrome

## Abstract

**Background:**

Tourette syndrome (TS) is associated with neuropsychiatric comorbidities, such as autism spectrum disorder (ASD), attention‐deficit/hyperactivity disorder (ADHD) and oppositional defiant disorder (ODD). Even though comorbidities are the main source of impairment in individuals with TS, family aggregation between TS and other neuropsychiatric disorders has been little explored. We therefore investigated associations of tic severity in probands with symptoms of ASD, ADHD, and ODD in their siblings and the influence of tic severity, age, and sex.

**Methods:**

The sample of the present study stems from the European Multicenter Tics in Children Study (EMTICS), a longitudinal observational study, with the present subsample of 196 probands with TS and their 220 full siblings (54.1% girls). We analyzed associations of probands’ tic severity with ASD, ADHD, and ODD symptoms in their siblings using generalized linear mixed‐effect negative binomial regression models.

**Results:**

Higher tic severity in probands was associated with higher scores of ASD symptoms in their siblings (IRR = 1.48, 95% confidence interval [95% CI] 1.03–2.12, *p* = 0.034); after excluding the three items in the Autism Spectrum Screening Questionnaire linked to stereotypies (that may be misinterpreted as tic‐like behaviors; IRR = 1.44 [95% CI] 0.99–2.09, *p* = 0.057) the effect size remained similar, yet reaching only near‐significance. Moreover, we demonstrated a significant interaction between probands’ tic severity and sex upon siblings’ symptoms of ADHD and ODD. Female siblings of probands with higher tic severity displayed more symptoms of ADHD and ODD, whereas this effect was absent in male siblings.

**Conclusions:**

This multicenter study demonstrated a link between probands’ current tic severity and siblings’ neuropsychiatric symptoms. Our study suggests a familial link between TS and ASD‐like symptoms, competencies as well as sex‐specific associations with ADHD and ODD symptoms in female siblings. The current study sheds light on a broader family tendency and highlights the need for targeted prevention in this vulnerable population. Our findings, however, call for further studies to better understand the genetic and environmental aggregation of influences between individuals with TS, ADHD, and ODD and their siblings.

## Graphical abstract

This multicenter study demonstrated that siblings of children with higher YGTSS score showed a higher level of ASD symptoms. We further report that only female siblings of children with high tic symptom severity showed higher inattention and ODD symptoms. In light of the limited literature regarding ASD, ODD, but also ADHD symptoms in relatives, our study provides a crucial addition by suggesting genetic patterns in clinical phenotyping, linking the severity of TS and ASD symptoms in first degree family members.

**Key points**

- Empirical data on neuropsychiatric symptoms in siblings of children with Tourette syndrome are largely absent, even though genetic and environmental aggregation of influences between individuals with Tourette syndrome, ADHD, and ODD and their siblings play an important role during development.
- This prospective study aimed to map the associations between tic severity in children with Tourette syndrome and clinical problems in their siblings, focused on ASD, ADHD and ODD.
- Our results indicate that higher tic severity in probands with Tourette syndrome was associated to higher scores of autism spectrum disorder symptoms in their siblings.
- Moreover, higher tic severity in probands was associated with higher ADHD and ODD symptoms in siblings that were girls, but not in boys.
- Our results highlight the need of regular monitoring of high‐risk children from preschool age onwards, taking sex differences into account.

## INTRODUCTION

Tourette syndrome (TS) is characterized by multiple motor tics and at least one vocal tic for at least 1 year, with a prevalence ranging from 0.32% to 0.85% and a 4‐fold higher risk in males compared to females (Knight et al., 2012; Scharf et al., 2015). TS may have a significant impact on the quality of life of affected children and their families. However, comorbidities rather than tics per se are often the main source of impairment (Claussen et al., 2018; Huisman‐van Dijk et al., 2019; Wolicki et al., 2019). Indeed, it is estimated that about 88% of individuals with TS in clinical populations have at least one comorbid condition (Ueda & Black, 2021), with attention‐deficit/hyperactivity disorder (ADHD) and obsessive‐compulsive disorder (OCD) affecting approximately 50%–60% and 30%–50% of patients, respectively (Browne et al., 2015; Hirschtritt et al., 2015; Huisman‐van Dijk et al., 2016; Tsetsos et al., 2016). Other less frequent comorbidities are autism spectrum disorder (ASD; present in 2.9%–20% of children with TS (Hirschtritt et al., 2015), and disruptive behavior disorders, such as oppositional defiant disorder (ODD) or conduct disorder, present in 30% of children with TS (Hirschtritt et al., 2015). Interestingly, males exhibit higher rates and more severe symptoms of ADHD and ASD, especially compared to females at younger age (Loomes et al., 2017; Mathews & Grados, 2011; Meoni et al., 2020), whereas females with TS are more prone to emotional problems (Garcia‐Delgar et al., 2022; Mathews & Grados, 2011; Meoni et al., 2020).

Comorbid disorders of TS, such as ADHD and ASD, have been demonstrated to be genetically related (Brainstorm et al., 2018; Cross‐Disorder Group of the Psychiatric Genomics Consortium, 2019; Jain et al., 2023; Yang et al., 2021) and represent extremes of dimensional traits that exist in the general population (Abdulkadir et al., 2019; Faraone & Larsson, 2019; Polderman et al., 2014; Stergiakouli et al., 2017; Yu et al., 2019). Indeed, previous Genome‐wide Complex Trait Analysis (GCTA) and family studies have shown a clinical overlap between these conditions, suggesting shared underlying genetic factors (Davis et al., 2013; Huisman‐van Dijk et al., 2016; Rizzo et al., 2017) that may as a consequence play out differently in siblings. Also, OCD has been shown to be genetically related to TS (Brainstorm et al., 2018; Mathews & Grados, 2011), with a population‐based study reporting a higher risk of OCD in siblings when the oldest sibling had TS (Browne et al., 2015). Notably, another family study found that first‐degree relatives of probands with TS and/or ADHD and OCD had an increased risk of co‐occurring ADHD and TS, which was linked to the presence of OCD in the proband (O'Rourke et al., 2011). Another study has shown that co‐existing ADHD + TS increased the risk of both comorbid ADHD + TS and TS in siblings (Roessner et al., 2016). Finally, a family study showed a higher rate of TS in relatives of probands with ADHD compared to controls, possibly reflecting some overlapping neurobiology or pathophysiology (Stewart et al., 2006).

However, despite the known comorbidity of TS with ADHD, ODD, and ASD within probands and familial aggregation, no studies have examined neuropsychiatric symptoms (ADHD, ODD, and ASD) in siblings of children with TS related to the proband’s tic severity. As tic severity correlates with a higher TS polygenic risk score (PRS (Yu et al., 2019)), bearing shared genetics with other neuropsychiatric symptoms (e.g., TS PRS weakly correlated with PRS of ADHD, ASD and OCD (Jain et al., 2023)), we propose that tic severity in probands would correlate with neuropsychiatric symptoms in their siblings possibly reflecting a shared genetic familial load (Mataix‐Cols et al., 2015; McGrath et al., 2014; Robertson, 2000).

In addition, a substantial body of literature suggests environmental influences in the association between proband’s psychiatric condition and sibling’s mental health problems (Jayasinghe et al., 2023) and the same seems even the case for parents (Hakulinen et al., 2024). Siblings growing up in the same family do not only share genetic but also environmental influences (e.g., family adversities), but they may also have unique experiences that shape their mental health outcomes differently, and one important factor of difference is the succinct influence of a sibling with a neurodevelopmental disorder (Wolff et al., 2022). More specifically, higher tic severity in probands may lead to higher sibling symptomatology (e.g., through increased family distress), as has been witnessed in other neuropsychiatric conditions (e.g., higher depressive symptoms, or cognitive alteration in siblings of patients with schizophrenia (Islam et al., 2018; Smith et al., 2016). In particular, children with ADHD and ODD symptoms may be susceptible to affective and emotion regulation problems (Cavanagh et al., 2017; Christiansen et al., 2019).

These unexplored facets necessitate further investigation, potentially highlighting the need for targeted prevention strategies tailored to this specific cohort of vulnerable children. Another topic in need of further investigation is the family aggregation of neuropsychiatric symptoms in relation to the sibling’s sex (Bolte et al., 2023; Joel & McCarthy, 2017), since neurodevelopmental conditions have a male preponderance in childhood (Brainstorm et al., 2018; Loomes et al., 2017; Mathews & Grados, 2011; Meoni et al., 2020; Schrag et al., 2019). A recent paper from our study group reported more severe tics in boys compared to girls (Garcia‐Delgar et al., 2022). Also given the different male‐female ratios of neurodevelopmental and disruptive disorders, sex‐specific family aggregation appears plausible. Still, concerning TS, previous studies did not find sex differences, neither in the familial risk and heritability (Mataix‐Cols et al., 2015) nor in tic severity (Meoni et al., 2020).

The aim of this study was to examine the association between current tic severity in children with TS (probands) and a range of tic related clinical problems (i.e., symptoms of ASD, ADHD, ODD, and OCD) in their siblings, adjusting for several potential confounders. We hypothesized that higher current tic severity in probands would be associated with increased neuropsychiatric symptoms in siblings due to shared genetic and environmental influences. We further aimed at exploring possible sex‐related differences, where we expected to find patterns in line with the sex‐specific distribution of neuropsychiatric disorders.

## METHODS

### Study sample

The sample of the present study stemmed from the European Multicenter Tics in Children Study (EMTICS), a longitudinal observational European multicenter study involving 16 clinical centers with collection of data taking place between 2013 and 2018. Detailed information about the study design and protocol has been described previously (Schrag et al., 2019). The EMTICS study comprises two cohorts: the COURSE cohort, including 715 youths aged 3–16 years with a chronic tic disorder (87.3% TS), and the ONSET cohort, including 260 children aged 3–10 years who are first‐degree relatives (thus, full siblings or children) of individuals with a chronic tic disorder, and who had never experienced tics, OCD, or trichotillomania, at the time of study entry. Exclusion criteria for both COURSE and ONSET cohorts encompassed children with serious medical/neurological illness and children or parents who were unable to comply with the protocol. For the present study, we only used data from the baseline evaluation of the COURSE and ONSET study, except for the information about possible onset of tics in the siblings [see also (Openneer et al., 2022)]. Of the 975 children from both cohorts who participated at baseline (see Supplementary Figure S1), 7 participants were excluded from the analyses due to breach inclusion (six probands from the COURSE cohort who had no chronic tic disorder; one participant from the ONSET cohort who had tics at study entry). Further, to focus on proband‐sibling pairs for our analyses, we excluded 552 participants from the pool of 968 participants. These exclusions were cases where a family had either only one member (*n* = 518 probands or unaffected children excluded), families with multiple probands but no unaffected siblings (*n* = 20 probands excluded), or families with only siblings and no probands (*n* = 14 participants from the ONSET cohort removed). The remaining sample included 416 participants, including 196 cases from the COURSE study who had at least one full sibling participating in the ONSET study (total number of siblings: *n* = 220) (Openneer et al., 2022). Of the 220 full siblings, 159 did not develop tics during the study, whereas 61 siblings did (see Supplementary Figure S1). Note that 7 children with OCD/trichotillomania represent protocol breaches yet being part of the database as to EMTICS policy. Further of note, within our analyzed participants, there were several families that consisted of either multiple siblings and one proband (*n* = 23) or multiple probands and one sibling (*n* = 5). Additionally, three families consisted of multiple siblings and multiple probands. Such probands or siblings were then part of more than one proband‐sibling pair. The Institutional Review Boards of the 16 clinical centers approved the study. All participants (parents and their child) provided written informed consent and/or assent.

### Measurements

The data were collected from parents and their children during a baseline hospital visit. Parents were asked to complete a series of questionnaires regarding their child’s neuropsychiatric symptoms. Siblingship was based on parent report.

#### Tic severity in probands

Severity of motor and vocal tics in probands was assessed by the clinician using the Yale Global Tic Severity Scale (YGTSS) (Leckman et al., 1989), the gold standard for the evaluation of tic severity, with good internal consistency and reliability (Storch et al., 2005). The YGTSS is a semi‐structured interview that evaluates the number, frequency, intensity, complexity, and interference of motor and vocal tics during the past week, with each domain scoring on a six‐point scale, ranging from 0 to 5 (Haas et al., 2021). A variable including the sum score for motor and vocal tics without impairment was created as the total tic severity scale score (range 0–50) (Haas et al., 2021).

#### Clinical measures in siblings

We collected information on symptom ratings of ASD, ADHD, and ODD in the unaffected siblings reported by the parents. We used the Autism Spectrum Screening Questionnaire (ASSQ) (Ehlers et al., 1999) to assess the severity of communication problems, social interaction, as well as restricted and repetitive behaviors. The questionnaire includes 27 items, allowing the creation of a total severity score (range 0–54; Cronbach’s *α* = 0.88 in our sample). Additionally, for sensitivity analyses, we created a restrictive sum score variable excluding the three items that target stereotypies, which may be confounded with tics (“Expresses sounds involuntarily; clears throat, grunts, smacks, cries or screams”; “Has involuntary face or body movements”; “Has clumsy, ill coordinated, ungainly, awkward movements or gestures”). To provide the prevalence in our sample, we considered a clinical cut‐off of 19 on the ASSQ representing the possible presence of high‐functioning ASD (Ehlers et al., 1999). Parents assessed the severity of ADHD and ODD symptoms during the last week, using 18 ADHD and 8 ODD items of the Swanson, Nolan and Pelham‐version IV rating scale (SNAP‐IV), representing items of the DSM‐IV TR (Association, 2000), scored on a four‐point scale. The SNAP‐IV showed a high inter‐reliability (ADHD: range 0–54, Cronbach’s *α* = 0.95; ODD: range 0–24, Cronbach’s *α* = 0.92) with three different sum score variables: (1) a sum score of the hyperactivity‐impulsivity scale, (2) a sum score of the inattention scale, and (3) a sum score of the oppositional defiant scale. To assess the severity of past week’s obsessive‐compulsive symptoms, the well‐validated semi‐structured interview Children’s Yale Brown Obsessive‐Compulsive Scale (CY‐BOCS (Goodman et al., 1989)) was used. The presence of OCD (CY‐BOCS) and ADHD diagnosis in probands and siblings was evaluated by well‐experienced clinicians performing clinical interviews according to DSM‐IV‐TR criteria (Schrag et al., 2019).

#### Covariates

Information on sociodemographic characteristics of the parents (education and ethnicity) and of the child (age and biological sex), use of psychotropic medication of the child, and perinatal history was collected through a standardized interview to the parents (Dietrich et al., 2015). Parental education was defined as the highest completed education level either of the mother or of the father, ranging from 1 to 6 (1 = “under 7 years of schooling”, 6 = “Post‐Graduate/Graduate/Professional Degree [MD, JD, PhD, MBA, MS/MA]). Ethnicity was dichotomized into “Caucasian” versus “Non‐Caucasian”. Medication use included antidepressants, mood stabilizers, and antipsychotics during the 2 weeks prior to the interview. Moreover, parents reported about their child’s weight and duration of pregnancy (Dietrich et al., 2015). We decided to use these variables because they are potentially associated with neuropsychiatric symptoms (Agrawal et al., 2018; Franz et al., 2018; Xia et al., 2021). Moreover, site information was included in every model to control for potential differences in recruitment and interview/questionnaire administration across the 16 clinical centers.

### Statistical analysis

Sample characteristics, prevalence rates for medication use, as well as perinatal history, and clinical characteristics were evaluated among probands (children from the COURSE cohort) and their siblings (ONSET cohort) by using Wilcoxon and Chi‐square tests to compare the differences between probands and their siblings for continuous and categorical variables, respectively. Associations between probands’ tic severity and the symptom dimensions in their siblings (symptom ratings of ASD, ADHD, and ODD) were determined using generalized linear mixed‐effect regression models with negative binomial link, including both fixed and random effects. The negative binomial link was chosen to accommodate the shape of the distribution of the outcome variables (right skewed, with a standard deviation greater than the mean). The fixed effect part encompassed the exposure of interest and other covariates that were controlled for in the model. The models included a random intercept to account for dependency across the observations from the members of the same family (i.e., siblings). For each outcome, the model included probands’ tic severity scores as the exposure of interest, adjusted for age and sex of the sibling, parental education, ethnicity, perinatal history, and psychotropic medication use. Further adjustment for site did not change the results, we therefore excluded it. Effect sizes were reported as incidence rate ratios (IRR). Moreover, to explore sex differences, we also added the interaction between the sex of the sibling and probands’ tic severity upon the outcome variables in the models, and subsequently tested the slopes in girls and boys. We were unable to use the data of the CY‐BOCS interview data for analyses because the majority of siblings reported no OCD symptoms, and we therefore encountered a distribution with a 0‐cell problem. The same problem occurred when we used a dichotomized variable for OCD symptoms. Note that we conducted our primary analyses in the total sample, including both children without and with a later tic onset ascertained during the study course, to make full use of the sample representing the values at the baseline assessment.

As additional sensitivity analyses, we repeated the same models in a subsample of siblings who did not develop tics to control for possible confounding by possibly higher baseline neuropsychiatric symptoms in a subgroup that later developed tics. Our previous study, comparing baseline clinical characteristics between children who did and did not develop tics, indicated higher neuropsychiatric symptom scores (e.g., conduct problems, ASD, and compulsions) preceding the onset of tics (Openneer et al., 2022). For transparency, we also present analyses regarding the subgroup of children who developed tics, despite the smaller sample size. Indeed, given the exclusive use of baseline data without knowing the subsequent development of tics in certain children over the study duration (as ascertained during the follow‐up assessments), we decided to include these participants in the primary analysis. However, we also present the outcomes without this subgroup of children who later developed tics. We also performed sensitivity analyses repeating all analyses by excluding potential confounding of the three items corresponding to stereotypical symptoms when calculating the association between tic severity in probands’ and siblings’ ASSQ sum score. Additionally, we ran the same models by excluding seven siblings with OCD at study entry, to control for potential sample selection bias due to the study protocol excluding siblings with OCD, which may have biased our results.

Statistical analyses were completed using the Statistical Analysis System (SAS Institute Inc., Cary, NC, USA), version 9.4 and *RStudio*, version R‐4.1.0. To fit the mixed models in the paper the lme4 (v 1.1.27.1) package from the *R* environment was used. A significance level of 0.05 was considered in all analyses.

## RESULTS

### Sample characteristics

For the sample characteristics of probands and their siblings see Table 1 and supplementary Table S1. Please note that for descriptive purposes Table 1 also contains comparisons between children from the ONSET study who did and did not develop tics. However, such a comparison was not an aim of our study as for the current analyses we had to select those children from the ONSET study who had siblings participating in the COURSE study. This resulted in a different sample and study methods, and therefore results deviate partially from our previous study that was specifically designed to compare clinical characteristics of children who did and did not later develop tics (Openneer et al., 2022).

**TABLE 1:** Sample characteristics of probands with a chronic tic disorder and their siblings at baseline (*n* = 416).

			Siblings (ONSET cohort)			Statistics ‐ *p*‐value^a^	
		Probands (COURSE cohort)	All siblings	Siblings who did not develop tics	Siblings who developed tics	Probands versus all siblings	Siblings who did versus who did not develop tics*
**N**		196	220	159	61	‐‐	‐‐
**Socio‐demographics**							
**Sex, % (n)**	**Female**	25.00 (49)	54.09 (119)	59.75 (95)	39.34 (24)	***<0.0001*** ^**b**^	***0.006*** ^**b**^
	**Male**	75.00 (147)	45.91 (101)	40.25 (64)	60.66 (37)		
**Age**	**Mean (sd)**	10.13 (2.59)	6.75 (2.15)	6.84 (2.24)	6.52 (1.92)	***<0.0001***	*0.338*
	**Range**	3.89–16.94	2.81–10.93	2.81–10.93	3.21–10.57		
**Ethnicity, % (n)**	**Caucasian**	76.53 (150)					
	**Other**	23.47 (46)					
**Parental education** ^**d**^, ^**e**^ **, % (n)**	**Under 7 years of schooling**	0.00 (0)					
	**Junior high school (7** ^**th**^ **‐9** ^**th**^ **grade)**	3.13 (6)					
	**GCSEs or high school diploma**	13.54 (26)					
	**A levels or 2 year college degree (associate degree)**	28.13 (54)					
	**Four year college/University degree (bachelor degree)**	32.29 (62)					
	**Post‐graduate/Graduate/Professional degree (MD, JD, PhD, MBA, MS/MA)**	21.92 (44)					
**Neuropsychiatric comorbidities**							
**ADHD**	**% (n)**	25.13 (49)	9.05 (19)	8.00 (12)	11.67 (7)	***<0.0001*** ^**b**^	*0.403* ^b^
**ASD**	**% (n)**	15.31 (30)	2.73 (6)	0.63 (1)	8.20 (5)	***<0.0001*** ^**c**^	***0.005*** ^**c**^
**Perinatal history**							
**Duration of pregnancy (weeks)**	**Mean (sd)**	39.10 (2.44)	38.84 (2.48)	38.71 (2.66)	39.15 (1.99)	*0.118*	*0.294*
	**Range**	26–46	24–44	24–44	32–44		
**Birth weight (kg)**	**Mean (sd)**	3.27 (0.61)	3.28 (0.58)	3.29 (0.59)	3.27 (0.56)	*0.743*	*0.999*
	**Range**	0.90–5.00	1.08–4.88	1.08–4.88	2.00–4.50		
**Psychotropic medication**							
**Medication use**	**% (n)**	30.77 (60)	3.20 (7)	3.16 (5)	3.285 (2)	***<0.0001*** ^**b**^	*1.000* ^c^
**Clinical characteristics**							
**Tic severity in probands (YGTSS total score)**	**Mean (sd)**	19.09 (8.90)					
	**Range**	0–43					
**Autism spectrum symptoms (ASSQ sum score)**	**Mean (sd)**	10.71 (9.55)	3.21 (5.97)	2.35 (3.77)	5.29 (9.10)	***<0.0001***	*0.069*
	**Range**	0–45	0–45	0–22	0–45		
**Autism spectrum symptoms (ASSQ without tic‐like items)**	**Mean (sd)**	7.72 (8.39)	2.87 (5.31)	2.14 (3.47)	4.64 (8.00)	***<0.0001***	*0.145*
	**Range**	0–39	0–38	0–20	0–38		
**Hyperactivity/Impulsivity symptoms (SNAP sum score)**	**Mean (sd)**	8.59 (6.88)	4.96 (6.13)	4.24 (5.66)	6.69 (6.90)	***<0.0001***	***0.008***
	**Range**	0–27	0–27	0–27	0–27		
**Inattention symptoms (SNAP sum score)**	**Mean (sd)**	10.55 (7.29)	4.92 (6.07)	4.59 (5.69)	5.71 (6.89)	***<0.0001***	*0.282*
	**Range**	0–27	0–27	0–27	0–27		
**ODD symptoms (SNAP sum score)**	**Mean (sd)**	8.52 (6.64)	4.30 (5.01)	3.76 (4.63)	5.62 (5.64)	***<0.0001***	***0.033***
	**Range**	0–24	0–24	0–24	0–20		
**OCD symptoms (CY‐BOCS sum score)**	**Mean (sd)**	6.73 (9.22)	1.05 (3.68)	0.87 (2.98)	1.54 (5.06)	***<0.0001***	*0.734*
	**Range**	0–36	0–24	0–17	0–24		

*Note*: Statistically significant results are in bold. p‐values are in italic. Bold‐italic values are therefore statistically significant p‐values.

Abbreviations: TD, Tourette disorder; YGTSS, Yale Global Tic Severity Scale; ASSQ, Autism Spectrum Screening Questionnaire; SNAP, Swanson, Nolan and Pelham rating scale; ADHD, Attention‐Deficit/Hyperactivity Disorder; ASD, Autism Spectrum Disorder; ODD, Oppositional Defiant Disorder; SDQ, Strengths and Difficulties Questionnaire.

^a^ Wilcoxon test

^b^ Chi‐square test

^c^ Fisher’s Exact test

^d^ Highest completed education level (higher score either of mother or father)

^e^ 10 missing.

* Note that this comparison was not an aim of our study as for the current analyses we had to select those children from the Onset study who had siblings participating in the Course study. This resulted in a different sample and therefore results deviating from our previous study that was specifically designed to compare clinical characteristics of children who did and did not later develop tics (11).

### Associations between tic severity in probands and symptoms of neuropsychiatric disorders in their siblings

Tic severity in probands was associated with neuropsychiatric symptoms in their siblings (Table 2). In particular, higher tic severity in probands was associated with higher ASSQ total scores in their siblings (Incidence Rate Ratio [IRR] = 1.48, 95% confidence interval [95% CI] 1.03–2.12, *p* = 0.034), indicating that siblings of probands with higher tic severity were having more communication problems, social interactions, and restricted/repetitive behaviors, compared with siblings of probands with lower tic severity. Adjusting for proband’s sex in the main models, or for the proband’s sex in interaction with the severity of tics did not change the association with neuropsychiatric symptoms in the siblings.

**TABLE 2:** Associations between tic severity in probands and symptoms of neuropsychiatric disorders in their siblings (*n* = 416).

	Sibling neuropsychiatric symptoms														
	ASSQ						SNAP								
	Sum score			Without stereotypy symptoms			Hyperactivity/impulsivity sum score			Inattention sum score			ODD sum score		
	IRR	95% CI	*p*‐value	IRR	95% CI	*p*‐value	IRR	95% CI	*p*‐value	IRR	95% CI	*p*‐value	IRR	95% CI	*p*‐value
Proband tic severity	**1.48**	**(1.03‐2.12)**	***0.034***	1.44	(0.99–2.09)	*0.057*	0.95	(0.73–1.23)	*0.677*	0.89	(0.69–1.15)	*0.361*	0.79	(0.61–1.03)	*0.079*
Proband tic severity * sex of the sibling	1.06	(0.67–1.67)	*0.805*	1.08	(0.68–1.71)	*0. 755*	**1.40**	**(1.03‐1.90)**	***0.032***	**1.55**	**(1.16‐2.07)**	***0.003***	**1.65**	**(1.19‐2.28)**	***0.002***
ICC (random effect)	0.77			0.75			0.81			0.82			0.77		
Number of observations	191			193			191			189			191		
Marginal *R* ^2^/Conditional *R* ^2^	0.10/0.79			0.09/0.78			0.18/0.85			0.22/0.86			0.14/0.80		

*Note*: Statistically significant results are in bold. p‐values are in italic. Bold‐italic values are therefore statistically significant p‐values.

Abbreviations: ASSQ, Autism Spectrum Screening Questionnaire; CI, Confidence Interval; ICC, Intraclass Correlation Coefficient; IRR, Incidence Rate Ratio; ODD, Oppositional Defiant Disorder; SNAP, Swanson, Nolan and Pelham rating scale.

Generalized linear mixed‐effect negative binomial regression models adjusted for sociodemographics (age, sex, ethnicity, parental education), perinatal history (duration of pregnancy, child’s weight), psychotropic medication use.

Incidence rate ratio is the estimated rate ratio for a one‐unit increase, given the other variables are held constant in the model.

Nine single siblings had two probands each from the same family and are therefore represented twice. Additionally, one family was composed by 2 probands and 5 siblings. The siblings are therefore represented twice.

**FIGURE 1:**
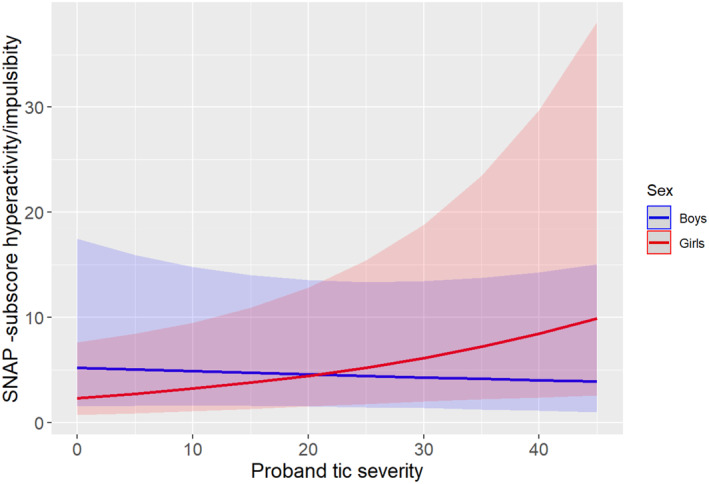
Interaction plot between the proband’s tic severity and the sex of the sibling upon the Swanson, Nolan and Pelham‐version IV rating scale (SNAP‐IV) hyperactivity/impulsivity severity score. For girls, there was a significant positive association between proband’s tic severity and the siblings’ hyperactivity/impulsivity symptoms. However, for boys, even if there is a decreasing slope, the association was not statistically significant.

**FIGURE 2:**
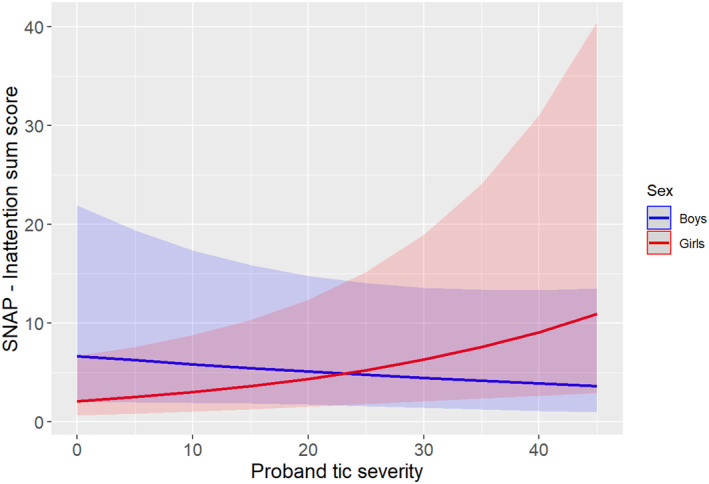
Interaction plot between the proband’s tic severity and the sex of the sibling upon the Swanson, Nolan and Pelham‐version IV rating scale (SNAP‐IV) inattention severity score. For girls, there was a significant positive association between proband’s tic severity and the siblings’ inattention symptoms. However, for boys, even if there is a decreasing slope, the association was not statistically significant.

**FIGURE 3:**
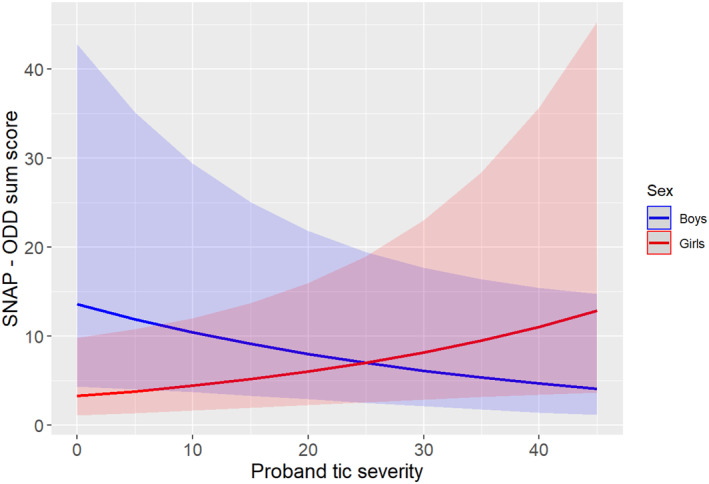
Interaction plot between the proband’s tic severity and the sex of the sibling upon the SNAP oppositional defiant disorder (ODD) severity score. For girls, there was a significant positive association between proband’s tic severity and the siblings’ ODD symptoms. However, for boys, even if there was a trend with a decreasing slope, the association was not statistically significant.

In contrast, there was no association (main effect) of tic severity in probands with ADHD (both inattention and hyperactivity‐impulsivity subscales) and ODD symptoms in their siblings. However, we found an interaction of tic severity of the proband with sex of the sibling on hyperactivity/impulsivity (IRR = 1.40, 95% CI 1.03–1.90, *p* = 0.032), inattention (IRR = 1.55, 95% CI 1.16–2.07, *p* = 0.003) and ODD (IRR = 1.65, 95% CI 1.19–2.28, *p* = 0.002) symptoms. Specifically, the slope for girls was significant, but the slope for boys was not (hyperactivity/impulsivity: IRR = 0.95, 95% CI 0.73–1.23, *p* = 0.677; inattention: IRR = 0.89, 95% CI 0.69–1.15, *p* = 0.361; ODD: IRR = 0.79, 95% CI 0.61–1.03, *p* = 0.079), indicating that female siblings of probands with higher tic severity had higher ADHD and ODD severity scores compared to female siblings of probands with lower tic severity, whereas no significant effects were found for boys, except a trend for ODD (see Table 2, as well as Figures 1, 2, 3). However, when we performed stratified analyses by sex, the models did not reach statistical significance, likely due to the smaller sample sizes (see Supplementary Table S2). Please note that undersampling of OCD in siblings did not allow us to investigate the full scope of OCD symptoms.

We performed sensitivity analyses using the same models for the association between tic severity in probands and ASSQ scores without the three items aiming to detect stereotypies in ASD, with the idea that these items could present confounders with respect to tics. Siblings of probands with higher tic severity did not show higher ASSQ total scores after excluding the three items concerning the stereotype movements (IRR = 1.44, 95% CI 0.99–2.09, *p* = 0.057). However, the effect size was similar to the one obtained without excluding the three stereotypy items, and the association was very close to be significant (see Table 2).

We also replicated the same analyses excluding the seven siblings with OCD, and the results were very similar (see Supplementary Table S3). This time, even after excluding the three items in the ASSQ linked to stereotypies (that may be misinterpreted as tic behaviors), the association between probands’ tic severity and ASSQ in siblings was still significant.

### Explorative associations between tic severity in probands and neuropsychiatric symptoms in their siblings who did and who did not have an onset of tics

First, when examining the associations between probands and the siblings who did not develop tics (Table 3 – Model 1), the association between proband tic severity and ASSQ scores in their siblings no longer reached statistical significance (IRR = 1.45, 95% CI 0.90–2.34, *p* = 0.128), however only with a small reduction of the IRR. We also did not find significant associations between probands’ tic severity and siblings’ neuropsychiatric symptoms in children who later developed tics (Table 3 – Model 2; specifically, for the association between probands’ tic severity and ASSQ score in siblings, IRR = 1.32, 95% CI 0.76–2.30, *p* = 0.332).

**TABLE 3:** Associations between tic severity in probands and symptoms of neuropsychiatric disorders in their siblings who did not (Model 1) and who did develop tics (Model 2).

		Sibling neuropsychiatric symptoms														
		ASSQ						SNAP								
		Sum score			Without stereotype symptoms			Hyperactivity/impulsivity sum score			Inattention sum score			ODD sum score		
		IRR	95% CI	*p*‐value	IRR	95% CI	*p*‐value	IRR	95% CI	*p*‐value	IRR	95% CI	*p*‐value	IRR	95% CI	*p*‐value
Model 1	Proband tic severity	1.45	(0.90–2.34)	*0.128*	1.37	(0.84–2.22)	*0.206*	0.85	(0.60–1.20)	*0.356*	0.76	(0.55–1.05)	*0.100*	0.72	(0.51–1.01)	*0.056*
	Proband tic severity * sex of the sibling	1.11	(0.63–1.96)	*0.717*	1.14	(0.64–2.02)	*0. 661*	**1.69**	**(1.15‐2.49)**	***0.008***	**1.85**	**(1.29‐2.66)**	***0.001***	**1.98**	**(1.31‐2.98)**	***0.001***
	ICC (random effect)	0.71			0.69			0.76			0.78			0.72		
	Number of observations	131			133			131			130			131		
	Marginal *R* ^2^/Conditional *R* ^2^	0.14/0.75			0.12/0.73			0.25/0.82			0.26/0.84			0.23/0.78		
Model 2	Proband tic severity	1.32	(0.76–2.30)	*0.332*	1.42	(0.79–2.54)	*0.237*	1.12	(0.77–1.63)	*0.545*	1.15	(0.76–1.73)	*0.512*	0.87	(0.58–1.31)	*0.513*
	Proband tic severity * sex of the sibling	1.25	(0.47–3.31)	*0.653*	1.22	(0.46–3.25)	*0.695*	0.86	(0.46–1.59)	*0.623*	1.09	(0.57–2.07)	*0.788*	1.07	(0.58–1.99)	*0.830*
	ICC (random effect)	0.84			0.83			0.84			0.82			0.81		
	Number of observations	60			60			60			59			60		
	Marginal *R* ^2^/Conditional *R* ^2^	0.13/0.86			0.12/0.85			0.08/0.85			0.09/0.83			0.11/0.83		

*Note*: Statistically significant results are in bold. p‐values are in italic. Bold‐italic values are therefore statistically significant p‐values.

Abbreviations: ASSQ, Autism Spectrum Screening Questionnaire; CI, Confidence Interval; ICC, Intraclass Correlation Coefficient; IRR, Incidence Rate Ratio; ODD, Oppositional Defiant Disorder; SNAP, Swanson, Nolan and Pelham rating scale.

Generalized linear mixed‐effect negative binomial regression models adjusted for sociodemographics (age, sex, ethnicity, parental education), perinatal history (duration of pregnancy, child’s weight), psychotropic medication use.

Incidence rate ratio is the estimated rate ratio for a one‐unit increase, given the other variables are held constant in the model.

Model 1: In the sample of siblings who did not develop tics (*n* = 159).

Model 2: In the sample of siblings who developed tics (*n* = 61; not adjusted for parental education).

Eight single siblings who did not develop tics had two probands each from the same family and are therefore represented twice.

Five single siblings who developed tics had two probands each from the same family and are therefore represented twice.

Second, in line with the results in the total sample, we found significant interactions of probands’ tic severity with sex, on hyperactivity‐impulsivity (IRR = 1.69, 95% CI 1.15–2.49, *p* = 0.008), inattention (IRR = 1.85, 95% CI 1.29–2.66, *p =* 0.001) and ODD symptom severity scores (IRR = 1.98, 95% CI 1.31–2.98, *p* = 0.001) in those siblings who did not develop tics. In line with our main findings, the slopes for girls were significant, but not for boys (hyperactivity/impulsivity: IRR = 0.85, 95%CI 0.60–1.20, *p* = 0.356; inattention: IRR = 0.76, 95% CI 0.55–1.05, *p* = 0.100; ODD: IRR = 0.72, 95% CI 0.51–1.01, *p* = 0.056). Female siblings of probands with higher tic severity had higher hyperactivity‐impulsivity, inattention and ODD symptoms compared with female siblings of probands with lower tic severity.

## DISCUSSION

The present study investigated the association between tic severity in probands and dimensional neuropsychiatric symptoms in their siblings, including ASD, ADHD, and ODD, using a large European sample of children and adolescents. Higher tic severity in probands with TS was associated with higher scores of ASD symptoms in their siblings compared with siblings of probands with lower tic severity. Although tic severity in probands was generally not associated with symptoms of ADHD and ODD in their siblings, we demonstrated an association between higher tic severity in the probands and higher ADHD and ODD symptoms in girls, but not in boys, irrespective of age and other potential confounders.

The association between tic severity in probands and higher ASD symptoms in siblings is a novel finding. The fact that we could not replicate this association in the subsample of siblings who did not develop tics later in the study is likely due to a decrease in statistical power resulting from the smaller sample size; note however that the magnitude of the IRR decreased only marginally and that the result approached statistical significance. Our result on the association between tic severity in probands and ASD symptoms in siblings failed to remain significant after excluding the three items asking for stereotypic behavior that may induce confounding with tics in the ASSQ. However, the result approached statistical significance with a similar effect size. This finding suggests a shared genetic predisposition of TS and ASD symptoms across siblings, or at least a sibling profile of impaired social and communicative competences. However, probands with TS often exhibit lower levels of self‐concept (Hanks et al., 2016), self‐esteem (Gill & Kompoliti, 2020; Silvestri et al., 2018), and social functioning (McGuire et al., 2013). Consequently, our finding may also be attributed to the presence of social impairments, rather than being solely indicative of ASD symptoms. Then again, factor analyses of the ASSQ have also pointed to items that typically pertain to ‘autistic traits’ less likely seen in normally developing children (e.g., formal speech and communication style (Junttila et al., 2023; Posserud et al., 2008). Previous studies have shown higher rates of ASD among children with TS, and higher concordance rates for ASD with tics, suggesting that there is a genetic link between TS and ASD (Brainstorm et al., 2018; Darrow et al., 2017; Lichtenstein et al., 2010). Additionally, a cross‐disorder genetic architecture assessment and systematic meta‐analysis of genome wide association studies revealed 11 new regions that displayed polymorphisms with a high probability of association with TS, ADHD, and ASD (Yang et al., 2021).

Our findings are consistent with those of a previous large population‐based study showing that siblings of children with ASD have an increased risk of neuropsychiatric disorders, including tic disorders (Jokiranta‐Olkoniemi et al., 2016). However, two major differences should be noted when comparing our study to the previous one. First, the exposure and outcome are reversed. The study in a population of children with ASD represents a very different profile of children compared to the children in the present study. Second, our study used a dimensional approach to neuropsychiatric symptoms, with ASD symptoms representing “social vulnerability” rather than a diagnosis, whereas in the cited study the authors included individuals with a diagnosis of ASD. In the present study, children did not receive high scores on the ASSQ questionnaire. Hence, we assume that the two samples have different profiles (Huisman‐van Dijk et al., 2016).

Concerning potential mechanisms linking TS and neuropsychiatric comorbidities, data from observational studies suggest that the dysfunction of interneurons in shared functional regions of the brain could lead to the development of ASD and ADHD (Carias & Wevrick, 2019; Clarke et al., 2012; Rapanelli et al., 2017). The main regions of the brain implicated in both ASD and TS are the cortico‐striatal circuits, which are considered as a common underlying link, relating self‐regulatory control with several neurodevelopmental disorders. Data from preclinical studies showed that specific inhibitory mechanisms, implicating GABAergic and cholinergic interneurons, are affected in both TS and ASD, implicating a potent molecular insight in pathogenetic overlap among TS and ASD (Lennington et al., 2016; Robertson et al., 2016). Additionally, a common underlying genetic factor has been suggested by previous genetic and family studies showing a clinical overlap between these conditions (Brainstorm et al., 2018; Davis et al., 2013; Huisman‐van Dijk et al., 2016; Rizzo et al., 2017).

Our observations that tic severity in probands was positively associated only in female siblings with ADHD (hyperactivity‐impulsivity, inattention) and ODD symptoms is a novel and somehow unexpected finding. It should be noted that our findings are independent of the proband’s sex, but not of the sibling’s sex. Our finding partially corroborates twin‐based and parent‐based studies that showed the familiality of TS, with transmission to offspring and correlation with ADHD (Brander et al., 2021; Zilhao et al., 2017). However, to our knowledge, no previous existing study examined sex differences in the familial transmission of ADHD and ODD in individuals with TS. Also, while ADHD and ODD are highly comorbid conditions (Comings et al., 1996), no evidence so far examined the link between tic expression, TS, and ODD symptoms in family or genetic studies. It is well‐documented that male children have greater rates of disruptive behavior disorders compared to females (Steel et al., 2014).

Whereas boys tend to display higher levels of hyperactivity and impulsivity, girls tend to exhibit more symptoms of inattention (Merikangas & Almasy, 2020). However, it is not always possible to distinguish symptoms of inattention from symptoms of emotional disorders, as they may be present in young girls with depression. Along the same lines, emotional problems and ODD symptoms were female‐specific precursors of tic onset and other neurodevelopmental disorders (e.g., ADHD) in our previous (Openneer et al., 2022) and other studies (Cavanagh et al., 2017; Rowe et al., 2010). This implicates a potent sex‐related association between hyperactivity‐impulsivity, inattention, ODD, and TS, in line with the sex‐specificity of familial aggregation in our present results. Additionally, the evidence of a familial link between TS and ADHD is still not clear (O'Rourke et al., 2011; Stewart et al., 2006). Previous studies have suggested a different genetic cause between TS and ADHD (Stewart et al., 2006), or that TS and comorbid attention problems (but not hyperactivity) represent a familial subtype (O'Rourke et al., 2011).

Although studies have indicated that environmental factors play a minor role in the association between TS and neuropsychiatric symptoms (Brander et al., 2021; Debes et al., 2010; Eysturoy et al., 2015; Heiman et al., 2020; Mataix‐Cols et al., 2015), parenting factors could affect the phenotypic appearance of a genetic predisposition (Zilhao et al., 2017). Parents may put less attention and care to the unaffected sibling, contributing to higher stress leading to psychopathological symptoms in the sibling. This may particularly pertain to emotion‐related symptoms (the reactive component of ODD symptoms) or ADHD symptoms in females being more vulnerable to emotional distress than males and in line with a prior family study that found a strong environmental correlation (and lack of genetic correlation) between TS and ADHD (Mathews & Grados, 2011).

Despite the importance of a genetic perspective that has been the prevailing one in the field of neuropsychiatry, we know that each individual’s mental health is influenced by a complex interplay of genetic, environmental, and social factors that are often difficult to measure and to determine. Siblings growing up in the same family may thus have shared experiences and exposures where a high symptom severity in probands may influence higher symptoms of ASD in the siblings, in line with a recent report showing that adolescents under stress are less prone to the processing of emotional and contextual information (Smith et al., 2020). Also the relationship between higher current tics in the proband and an increment in female siblings for symptoms of ODD and ADHD may be mediated by the stress (Isaksson et al., 2015) induced in the family environment due to tics and requires more exploration. Of note, a recent study reported that girls with ADHD experienced a high level of perceived stress during adolescence than their male counterparts or girls without ADHD (Frick et al., 2023).

Enhanced knowledge of family constellations in these neuropsychiatric vulnerabilities could indicate that the concept of “ESSENCE” (Gillberg, 2010) could possibly be extended to a family constellation. ESSENCE (early symptomatic syndromes eliciting neurodevelopmental clinical examinations) assumes that comorbidity is almost always present in developmental disorders, that it is not always possible to decide which diagnosis is correct, and that ADHD or ASD may be diagnosed at different developmental stages. Therefore, in families, the neuropsychiatric condition of one of the children calls for increased attention to the siblings to rule out certain developmental problems or to support the development of a child with specific interventions (Gillberg, 2010).

### Strengths and limitations

The present study is the first to establish the familial aggregation of neuropsychiatric symptoms in siblings of probands with TS, adjusting for potential confounders in a large international sample. Despite these strengths, several limitations of the present study should be noted. First, this was a sibling‐based study with TS‐affected probands recruited in specialized clinics and by patient organizations for the purpose of participating in a research study, and those recruited may not be fully representative of individuals with tic disorders in the general population. Second, we only performed cross‐sectional analyses. Third, the exclusion of siblings with OCD in the EMTICS study may have underestimated the prevalence of siblings with neuropsychiatric symptoms in our sample and we were thus not able to investigate links between probands’ tic severity and severity of OCD symptoms in their siblings. Fourth, the evaluation of probands’ tic severity was based on the gold standard instrument that reports tics during the past week, which may not accurately reflect lifetime tic severity. However this instrument is regarded the most comprehensive, reliable, and valid instrument rating tic severity (Martino et al., 2017). A measurement limited to the last week could potentially introduce bias, in the sense of an underestimation of potential effects of tic‐symptoms, when assessing the impact of proband tic severity on the occurrence and severity of neuropsychiatric symptoms in siblings. Similarly, assessments of autism, hyperactivity/inattention, and ODD symptoms were not all conducted in the same time frame as the evaluation of tic severity. Additionally, it is important to note that some assessments relied on self‐reported measures, and others on clinician‐rated ones. The presence of informant discrepancies, coupled with variations in the assessment timeframe, could potentially have impacted the findings. Fifth, probands differed significantly from their siblings with respect to sex and age, which could affect our results. Sixth, our study design does not allow to identify genetic versus environmental contributions. Future, large population‐based studies addressing these limitations are needed.

## CONCLUSIONS AND FURTHER PERSPECTIVES

This multicenter study demonstrated that siblings of children with higher YGTSS score showed a higher level of ASD symptoms. We further report that only female siblings of children with high tic symptom severity showed higher ADHD and ODD symptoms. In light of the limited literature regarding ASD, ODD, but also ADHD symptoms in relatives, our study provides a crucial addition by suggesting genetic patterns in clinical phenotyping, linking the severity of TS and ASD symptoms in first degree family members. Further studies are necessary to elucidate the associations between children with TS and ADHD and ODD in siblings. This opens a perspective on a larger familial aggregation that calls for selective prevention in this group of vulnerable children. It is important to investigate whether specific interventions in the child affected with TS or in the whole family could prevent the expression of emotional, behavioral, and tics in unaffected siblings.

This study emphasizes the importance of increasing awareness for the siblings of children with TS, who have a higher risk of developing not only TS, but also other neuropsychiatric symptoms and an exposure to a higher level of stress in the family. Even though the siblings identified in this study are not necessarily suffering from a veritable ASD, their profile prompts early interventions that could strengthen their social competences.

## AUTHOR CONTRIBUTIONS

**Olga Sidiropoulou**: Conceptualization; Investigation; Methodology; Writing – original draft; Writing – review & editing. **Jennifer Glaus**: Conceptualization; Formal analysis; Methodology; Project administration; Supervision; Writing – original draft; Writing – review & editing. **Julie Hagstrøm**: Conceptualization; Investigation; Methodology; Writing – review & editing. **Setareh Ranjbar**: Conceptualization; Formal analysis; Methodology; Writing – review & editing. **Renata Rizzo**: Conceptualization; Investigation; Methodology; Writing – review & editing. **Pieter J. Hoekstra**: Conceptualization; Formal analysis; Funding acquisition; Investigation; Methodology; Resources; Writing – review & editing. **Andrea Dietrich**: Conceptualization; Formal analysis; Funding acquisition; Investigation; Methodology; Project administration; Supervision; Writing – original draft; Writing – review & editing. **Kerstin J. Plessen**: Conceptualization; Formal analysis; Funding acquisition; Investigation; Methodology; Project administration; Supervision; Writing – original draft; Writing – review & editing.

## CONFLICT OF INTEREST STATEMENT

The authors have no conflicts of interest to declare.

## ETHICAL CONSIDERATIONS

This study was approved by the institutional review boards at all participating clinical centers in accordance with the ethical standards laid down in the 1964 Declaration of Helsinki and its later amendments. All parents (or the legal guardian) provided written informed consent and the participating child written or oral assent before entering the study.

## Supporting information

Supporting Information S1

## Data Availability

De‐identified participant data related to all demographic, clinical, and laboratory variables will be shared following request made by any qualified investigators to the study authors.
