# Supplementary material for: Neuropsychiatric symptoms in siblings of children with Tourette syndrome in the EMTICS study
Source: JCPP Adv. 2024 Sep 20;5(3):e12277. doi: 10.1002/jcv2.12277 (PMC12446703; doi:10.1002/jcv2.12277)
Supplement: Supplementary file 1 — Supporting Information S1 [file JCV2-5-e12277-s001.docx]

**Supporting Information**

**Neuropsychiatric symptoms in siblings of children with Tourette syndrome: the EMTICS study**

Sidiropoulou O. , Glaus J., Hagstrøm J., Ranjbar S., Rizzo R., EMTICS collaborative group, Hoekstra P.J., Dietrich A., Plessen K.J.

**Appendix S1. EMTICS group authorship (contributing authors)***

EMTICS group members are **Alan Apter,MD,^1^ Valentina Baglioni, MD,^2^ Juliane Ball, PhD,^3^ Noa Benaroya-Milshtein, MD, PhD,^1^ Emese Bognar, MSc,^4,5^ Bianka Burger, MSc,^6,7^ Judith Buse, PhD,^8^ Francesco Cardona, MD,^2^ Marta Correa Vela, MD,^9^ Andrea Dietrich, PhD,^10^ Carolin Fremer, MSc,^11^ Blanca Garcia-Delgar, MD,^12^ Julie Hagstrøm, PhD,^13^ Tammy J. Hedderly, MD,^14^ Isobel Heyman, MD, PhD,^15^ Pieter J. Hoekstra, MD, PhD,^10^ Chaim Huyser, MD, PhD,^16,17^ Marcos Madruga-Garrido, MD,^18^ Anna Marotta,^19^ Davide Martino, MD, PhD,^20^ Pablo Mir, MD, PhD,^9,21^ Astrid Morer Liñan, MD, PhD,^12,22,23^ Norbert Müller, MD, PhD,^6^ Kirsten Müller-Vahl, MD,^11^ Alexander Münchau, MD,^24^ Peter Nagy, MD,^4,5^ Valeria Neri, MD,^2^ Alessandra Pellico, MD,^25^ Kerstin J. Plessen, MD, PhD,^26^ Cesare Porcelli, MD,^19^ Renata Rizzo, MD, PhD,^25^ Veit Roessner, MD, PhD,^8^ Daphna Ruhrman, PhD,^1^ Jaana M.L. Schnell, MSc,^6^ Anette Schrag, PhD, FRCP,^27^ Paola Rosaria Silvestri, MD,^2^ Liselotte Skov, MD, DMSc,^28^ Tamar Steinberg, MD,^1^ Friederike Tagwerker Gloor, MSc,^3^ Zsanett Tarnok, PhD,^4^** **Susanne Walitza, MD,^3^** **Elif Weidinger, MD, PhD.^6^**

^1^Child and Adolescent Psychiatry Department, Schneider Children's Medical Center of Israel, affiliated to Sackler Faculty of Medicine, Tel Aviv University, Petah-Tikva, Israel

^2^University La Sapienza of Rome, Department of Human Neurosciences, Rome, Italy

^3^Department of Child and Adolescent Psychiatry and Psychotherapy, University of Zurich, Zurich, Switzerland

^4^Vadaskert Child and Adolescent Psychiatric Hospital, Budapest, Hungary

^5^Semmelweis University, Budapest, Hungary

^6^Department of Psychiatry and Psychotherapy, University Hospital, LMU Munich, Munich, Germany

^7^Marion von Tessin Memory-Zentrum GmbH, Munich, Germany

^8^Department of Child and Adolescent Psychiatry, Faculty of Medicine of the TU Dresden, Dresden, Germany

^9^Unidad de Trastornos del Movimiento. Instituto de Biomedicina de Sevilla (IBiS). Hospital Universitario Virgen del Rocío/CSIC/Universidad de Sevilla. Seville, Spain.

^10^University of Groningen, University Medical Center Groningen, Department of Child and Adolescent Psychiatry, Groningen, The Netherlands

^11^Clinic of Psychiatry, Socialpsychiatry and Psychotherapy, Hannover Medical School, Hannover, Germany

^12^Department of Child and Adolescent Psychiatry and Psychology, Institute of Neurosciences, Hospital Clinic Universitari, Barcelona, Spain

^13^Child and Adolescent Mental Health Center, Mental Health Services, Capital Region of Denmark, Denmark

^14^Evelina London Children’s Hospital GSTT, Kings Health Partners AHSC, London, UK

^15^Great Ormond Street Hospital for Children, and UCL Institute of Child Health, London, UK

^16^Levvel, Academic Center for Child and Adolescent Psychiatry, Amsterdam, The Netherlands

^17^Amsterdam UMC, Department of Child and Adolescent Psychiatry, Amsterdam, The Netherlands

^18^Neuropediatrics, Centro de Pediatría de Sevilla, Hospital Viamed Santa Ángela De la Cruz, Seville,

Spain

^19^Azienda Sanitaria Locale di Bari, Mental Health Department, Child and Adolescent Service of Bari Metropolitan Area, Bari, Italy

^20^Department of Clinical Neurosciences, University of Calgary, Calgary, Canada

^21^Centro de Investigación Biomédica en Red sobre Enfermedades Neurodegenerativas (CIBERNED), Madrid, Spain

^22^Institut d'Investigacions Biomediques August Pi i Sunyer (IDIBAPS), Barcelona, Spain

^23^Centro de Investigacion en Red de Salud Mental (CIBERSAM), Instituto Carlos III, Spain

^24^Institute of Systems Motor Science, Center of Brain, Behavior and Metabolism, University of Lübeck, Lübeck, Germany

^25^Child Neuropsychiatry Section, Department of Clinical and Experimental Medicine, School of Medicine, Catania University, Catania, Italy

^26^Division of Child and Adolescent Psychiatry, Department of Psychiatry, University Medical Center, University of Lausanne, Lausanne, Switzerland

^27^Department of Clinical Neuroscience, UCL Institute of Neurology, University College London, London, UK

^28^Paediatric Department, Herlev University Hospital, Herlev, Denmark

**Appendix S2. Full description of Acknowledgements**

The authors are deeply grateful to all children and their parents who willingly participated and made this research possible. This project has received funding from the European Union’s Seventh Framework Programme for research, technological development, and demonstration under Grant agreement no. 278367.

This research was further supported by the National Institute for Health Research Biomedical Research Centre at Great Ormond Street Hospital for Children NHS Foundation Trust and University College London (Heyman); the Guys and St Thomas' NHS Foundation Trust (Hedderly, Turner); the Spanish Ministry of Science and Innovation [RTC2019-007150-1], the Instituto de Salud Carlos III-Fondo Europeo de Desarrollo Regional [ISCIII-FEDER] [PI14/01823, PI16/01575, PI18/01898, PI19/01576], the Consejería de Economía, Innovación, Ciencia y Empleo de la Junta de Andalucía [CVI-02526, CTS-7685], the Consejería de Salud y Bienestar Social de la Junta de Andalucía [PI-0471-2013, PE-0210-2018, PI-0459-2018, PE-0186-2019], and the Fundación Alicia Koplowitz (Mir); the EU [FP7-HEALTH-2011No. 278367, FP7-PEOPLE-2012-ITN No. 316978] (Fremer and Müller-Vahl); the German Research Foundation [DFG: GZ MU 1527/3-1], the German Ministry of Education and Research [BMBF: 01KG1421], the National Institute of Mental Health [NIMH], and the Tourette Gesellschaft Deutschland e.V., the Else-Kroner-Fresenius-Stiftung (Müller-Vahl); the Possehl-Stiftung [Lübeck, Germany], Margot und Jürgen Wessel Stiftung [Lübeck, Germany], Tourette Syndrome Association [Germany], Interessenverband Tourette Syndrom [Germany], CHDI, Damp-Stiftung [Kiel, Germany], Academic research support: Deutsche Forschungsgemeinschaft (DFG): projects 1692/3-1, 4-1, SFB 936, and FOR 2698 [project numbers 396914663, 396577296, 396474989]; and European Reference Network – Rare Neurological Diseases [ERN – RND; Project ID No 739510] (Münchau); and the National Institute for Health Research UCLH Biomedical Research Centre (Schrag).

We thank all colleagues at the various study centres who contributed to data collection: Judy Grejsen (Paediatric Department, Herlev University Hospital, Herlev, Denmark); Stephanie Enghardt (TU Dresden, Germany); Stefanie Bokemeyer and Cornelia Reichert (MHH Hannover, Germany); Jenny Schmalfeld (Lübeck University, Germany); Elif Weidinger, Bianka Burger (LMU Munich, Germany); Martin L. Woods (Evelina London Children’s Hospital, United Kingdom); Franciska Gergye, Margit Kovacs, and Reka Vidomusz (Vadaskert Budapest, Hungary); Silvana Fennig, Ella Gev, Matan Nahon, Danny Horesh, Chen Regev, and Tomer Simcha, (Tel Aviv, Petah-Tikva, Israel); Marieke Messchendorp, Thaïra J.C. Openneer, Frank Visscher, and the Stichting Gilles de la Tourette (UMCG Groningen, Netherlands); Maria Teresa Cáceres, Fátima Carrillo, Laura Vargas, and Ángela Periañez Vasco (Seville, Spain); Marina Redondo (FRCB, Barcelona, Spain); Anna Marotta (ASL, Bari, Italy); Alessandra Pellico (Catania, Italy); Paola Rosaria Silvestri (Rome, Italy); Annelieke Hagen (De Bascule and Academic Medical Center, Amsterdam, the Netherlands), and all who may not have been mentioned.

**Appendix S3. Figure S1: Flow chart**

**
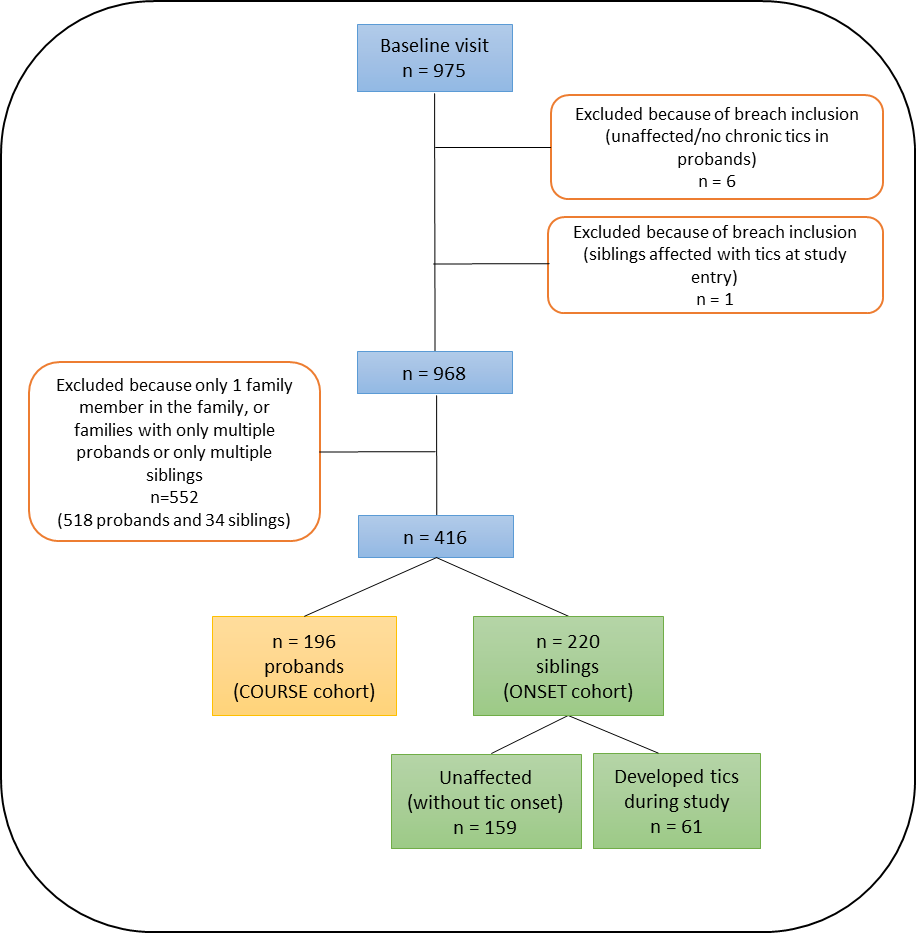
Figure S1:** Flow chart. Participants were excluded in case there was no proband-sibling pair, with (a) n=518 only 1 family member (n=493 probands and n=25 siblings), (b) n=10 only multiple probands, and (c) n=7 only multiple siblings. Note that the EMTICS study did not exclude children with obsessive-compulsive disorder (OCD) and trichotillomania from the ONSET cohort, representing protocol breaches to the exclusion criteria, (in the present subsample, n = 7).

**Supplementary Table S1: Sample characteristics of probands with a chronic tic disorder and their siblings at baseline stratified by sex (n=416)**

|  | |  | **Probands (COURSE cohort)** | | |  | **All Siblings** | | |
| --- | --- | --- | --- | --- | --- | --- | --- | --- | --- |
|  |  |  | **Girls** |  | **Boys** |  | **Girls** |  | **Boys** |
| **N** |  |  | 49 |  | 147 |  | 119 |  | 101 |
| **Clinical characteristics** | |  |  |  |  |  |  |  |  |
| **Tic severity in probands (YGTSS total score)** | **Mean (sd)** |  | 17.76 (9.48) |  | 19.53 (8.68) |  |  |  |  |
|  | **Range** |  | 0-42 |  | 0-43 |  |  |  |  |
| **Autism spectrum symptoms (ASSQ sum score)** | **Mean (sd)** |  | 10.47 (8.88) |  | 10.80 (9.80) |  | 2.81 (4.70) |  | 3.68 (7.21) |
|  | **Range** |  | 0-33 |  | 0-45 |  | 0-25 |  | 0-45 |
| **Autism spectrum symptoms (ASSQ without tic-like items)** | **Mean (sd)** |  | 7.75 (7.98) |  | 7.71 (8.56) |  | 2.51 (4.04) |  | 3.29 (6.50) |
|  | **Range** |  | 0-27 |  | 0-39 |  | 0-20 |  | 0-38 |
| **Hyperactivity/Impulsivity symptoms (SNAP sum score)** | **Mean (sd)** |  | 7.41 (6.68) |  | 9.01 (6.93) |  | 4.44 (5.18) |  | 5.59 (7.09) |
|  | **Range** |  | 0-24 |  | 0-27 |  | 0-24 |  | 0-27 |
| **Inattention symptoms (SNAP sum score)** | **Mean (sd)** |  | 10.67 (7.78) |  | 10.51 (7.14) |  | 4.33 (5.42) |  | 5.62 (6.72) |
|  | **Range** |  | 0-26 |  | 0-27 |  | 0-27 |  | 0-27 |
| **ODD symptoms (SNAP sum score)** | **Mean (sd)** |  | 8.57 (6.90) |  | 8.50 (6.58) |  | 3.90(4.58) |  | 4.79 (5.46) |
|  | **Range** |  | 0-24 |  | 0-24 |  | 0-19 |  | 0-24 |
| **OCD symptoms (CY-BOCS sum score)** | **Mean (sd)** |  | 5.73 (8.72) |  | 7.06 (9.39) |  | 0.72 (3.13) |  | 1.45 (4.22) |
|  | **Range** |  | 0-36 |  | 0-36 |  | 0-24 |  | 0-19 |

**Supplementary Table S2: Associations between tic severity in probands and symptoms of neuropsychiatric disorders in their siblings for boys and girls separately (n=416).**

|  |  | **Sibling neuropsychiatric symptoms** | | | | | | | | | | |
| --- | --- | --- | --- | --- | --- | --- | --- | --- | --- | --- | --- | --- |
|  |  | **SNAP** | | | | | | | | | | |
|  |  | **Hyperactivity/Impulsivity** | | |  | **Inattention sum score** | | |  | **ODD sum score** | | |
|  |  | **IRR** | **95% CI** | ***p*-value** |  | **IRR** | **95% CI** | ***p*-value** |  | **IRR** | **95% CI** | ***p*-value** |
| **Boys** | **Proband tic severity** | 1.05 | 0.76-1.44 | 0.785 |  | 0.97 | (0.71-1.33) | *0.869* |  | 0.81 | (0.59-1.10) | *0.181* |
|  | **ICC (random effect)** | 0.84 | | | | 0.83 | | |  | 0.80 | | |
|  | **Number of observations** | 86 | | | | 86 | | |  | 86 | | |
|  | **Marginal R^2^ / Conditional R^2^** | 0.10/0.86 | | | | 0.13 / 0.85 | | |  | 0.09 / 0.82 | | |
|  |  |  | | | |  | | |  |  | | |
| **Girls** | **Proband tic severity** | 1.14 | 0.88-1.48 | 0.311 |  | 1.27 | (0.96-1.67) | *0.092* |  | 1.23 | (0.95-1.58) | *0.112* |
|  | **ICC (random effect)** | 0.75 | | | | 0.76 | | |  | 0.72 | | |
|  | **Number of observations** | 105 | | | | 103 | | |  | 105 | | |
|  | **Marginal R^2^ / Conditional R^2^** | 0.26/0.81 | | | | 0.26 / 0.82 | | |  | 0.19 / 0.78 | | |

*Abbreviations*: ASSQ, Autism Spectrum Screening Questionnaire; CI, Confidence Interval; IRR, Incidence Rate Ratio; ODD, Oppositional Defiant Disorder; SNAP, Swanson, Nolan and Pelham rating scale.

Generalized linear mixed-effect negative binomial regression models adjusted for sociodemographics (age, sex, ethnicity, parental education), perinatal history (duration of pregnancy, child’s weight), psychotropic medication use.

Incidence rate ratio is the estimated rate ratio for a one-unit increase, given the other variables are held constant in the model.

Statistically significant results are in bold. *p*-values are in italic.

Six boy siblings had two probands from the same family and are therefore represented twice.

Seven girl siblings had two probands from the same family and are therefore represented twice.

**Supplementary Table S3.** **Associations between tic severity in probands and symptoms of neuropsychiatric disorders in their siblings excluding 7 siblings with OCD (n=404)**

| **Sibling neuropsychiatric symptoms** | | | | | | | | | | | | | | | | | | | |
| --- | --- | --- | --- | --- | --- | --- | --- | --- | --- | --- | --- | --- | --- | --- | --- | --- | --- | --- | --- |
|  | **ASSQ** | | | | | | |  | **SNAP** | | | | | | | | | | |
|  | **Sum score** | | |  | **Without stereotype symptoms** | | |  | **Hyperactivity/ impulsivity sum score** | | |  | **Inattention sum score** | | |  | **ODD sum score** | | |
|  | **IRR** | **95% CI** | ***p*-value** |  | **IRR** | **95% CI** | ***p*-value** |  | **IRR** | **95% CI** | ***p*-value** |  | **IRR** | **95% CI** | ***p*-value** |  | **IRR** | **95% CI** | ***p*-value** |
| **Proband tic severity** | **1.65** | **(1.13-2.41)** | ***0.010*** |  | **1.59** | **(1.07-2.35)** | ***0.022*** |  | 1.01 | (0.77-1.32) | *0.929* |  | 0.88 | (0.67-1.15) | *0.356* |  | 0.80 | (0.61-1.06) | *0.116* |
| **Proband tic severity * sex of the sibling** | 0.93 | (0.59-1.49) | *0.771* |  | 0.95 | (0.59-1.54) | *0. 847* |  | 1.33 | (0.97-1.82) | *0.081* |  | **1.64** | **(1.21-2.21)** | ***0.001*** |  | **1.63** | **(1.17-2.28)** | ***0.004*** |
| **ICC (random effect)** | 0.75 | | |  | 0.73 | | |  | 0.80 | | |  | 0.80 | | |  | 0.77 | | |
| **Number of observations** | 182 | | |  | 184 | | |  | 181 | | |  | 179 | | |  | 184 | | |
| **Marginal R^2^ / Conditional R^2^** | 0.11 / 0.78 | | |  | 0.10 / 0.76 | | |  | 0.18 / 0.84 | | |  | 0.20 / 0.84 | | |  | 0.14 / 0.81 | | |

*Abbreviations*: ASSQ, Autism Spectrum Screening Questionnaire; CI, Confidence Interval; ICC, Intraclass Correlation Coefficient; IRR, Incidence Rate Ratio; ODD, Oppositional Defiant Disorder; SNAP, Swanson, Nolan and Pelham rating scale.

Generalized linear mixed-effect negative binomial regression models adjusted for sociodemographic (age, sex, ethnicity, parental education), perinatal history (duration of pregnancy, child’s weight), and psychotropic medication use.

Incidence rate ratio is the estimated rate ratio for a one-unit increase, given the other variables are held constant in the model.

Statistically significant results are in bold. *p*-values are in italic.

Ten single siblings had two probands each from the same family and are therefore represented twice.
